# The Role of LINC01564, RAMS11, CBX4 and TOP2A in Hepatocellular Carcinoma

**DOI:** 10.3390/biomedicines11010056

**Published:** 2022-12-26

**Authors:** Eman A. E. Badr, Elshaymaa I. Elmongy, Rasha Galal Mostafa, Ibrahim El-Tantawy El-Sayed, Abd El-Naser Abd El-Ati Gad Allah, Asmaa Khairy Ahmed, Yasser A. S. Elghobashy

**Affiliations:** 1 Department of Medical Biochemistry and Molecular Biology, Faculty of Medicine, Menoufia University, Shebeen El-Kom 32511, Egypt; 2 Department of Pharmaceutical Sciences, College of Pharmacy, Princess Nourah bint Abdulrahman University, P.O. Box 84428, Riyadh 11671, Saudi Arabia; 3 Department of Medical Microbiology and Immunology, Faculty of medicine, Menoufia University, Shebeen El-Kom 32511, Egypt; 4 Department of Chemistry, Faculty of Science, Menoufia University, Shebeen El-Kom 32511, Egypt; 5 Department of Internal Medicine, Faculty of Medicine, Menoufia University, Shebeen El-Kom 32511, Egypt

**Keywords:** lncRNA, chronic hepatitis C, CBX4, LINC01564, RAMS11 and TOP2A, HCC

## Abstract

Background: Hepatocellular carcinoma (HCC) is the most common histologic type of primary liver cancers worldwide. Hepatitis C virus (HCV) infection remains a major risk factor for chronic liver disease, cirrhosis, and HCC. To understand the molecular pathogenesis of HCC in chronic HCV infection, many molecular markers are extensively studied, including long noncoding RNAs (lncRNA). Objective: To evaluate the expression levels of lncRNAs (LINC01564, RAMS11), CBX4, and TOP2A in patients with chronic HCV infection and patients with HCC on top of chronic HCV infection and correlate these levels with the clinicopathological features of HCC. Subjects and Methods: One hundred and fifty subjects were enrolled in this study and divided into three groups: group I included 50 patients with HCC on top of chronic hepatitis C (CHC), group II included 50 patients with CHC only, and group III included 50 healthy individuals as a control group. LncRNAs relative expression level was determined by RT-PCR. Results: lncRNA (LINC01564, RAMS11), CBX4, and TOP2A relative expression levels were upregulated in both patient groups compared to controls (*p* < 0.001*), with the highest levels in the HCC group compared with the CHC group. Additionally, these levels were significantly positively correlated with the clinicopathological features of HCC. Conclusions: The lncRNA (LINC01564, RAMS11), CBX4, and TOP2A relative expression levels were upregulated in CHC patients—in particular, patients with HCC. Thus, these circulatory lncRNAs may be able to serve as promising noninvasive diagnostic markers for HCC associated with viral C hepatitis.

## 1. Introduction

HCC is the most prevalent, among primary liver cancers and the third leading cause of cancer-related deaths worldwide [1]. In Egypt, liver cancer accounts for 1.68% of all cancer cases, with 70.48% of these cancers caused by HCC. HCC represents the main complication of cirrhosis [2]. It is characterized by having a poor prognosis and survival rate caused by the high recurrence rate, metastasis after surgical resection, and resistance to standard chemotherapy and radiotherapy [3]. Chronic hepatitis B and/or C infections in particular can hasten the onset of HCC by inciting the body’s immune system to attack the liver cells, some of which are infected with the virus while others are merely bystanders [4]. Free radicals, such as reactive oxygen species and nitric oxide reactive species, are released by activated immune system inflammatory cells, which cause DNA damage and result in carcinogenic gene mutations [5]. Despite improvements in disease diagnosis and clinical therapy, as well as knowledge of risk factors for the development of HCC, the molecular mechanisms underlying hepatocarcinogenesis are still poorly understood. Recent experimental studies have revealed that long non-coding RNAs (lncRNAs) have a significant role in the etiology of many human disorders. A very active area of study on the role of lncRNAs in the initiation, development, and metastasis of HCC has evolved during the past ten years [6]. Long non-coding RNAs (LncRNAs) are transcripts longer than 200 nucleotides but not translated into proteins [7], and the majority of them are highly expressed in differentiated tissues or certain cancer types [8]. LncRNAs influence a number of biological processes, including differentiation, development, and biogenesis, as well as a variety of human disorders, including certain malignancies [9,10]. Exosomes are considered one of the mechanisms that explain the role of LncRNAs in hepatocarcinogenesis [11]. The exosomes are membrane vesicle structures with 30 –100 nm in size, originating from the endosomes and secreted from almost all cells [12]. Evidence indicates that proteins, DNAs and various forms of RNA, including, long noncoding RNA (lncRNA), transferred by exosomes, contribute to the development of HCC, and many other diseases [13,14,15,16]. It was reported that cancer-derived exosomes mediate HCC progression through enhancing cell proliferation [17], increasing epithelial-mesenchymal transition (EMT) [18] and angiogenesis [19]. They play a critical role in immunomodulators which may cause a strong immune response [20].

It was found that lncRNA, such as ROR, VLDLR, and FAL1, was enriched in HCC-derived exosomes and plays a role in the modulation of hepatoma cellular responses to sorafenib, chemoresistance, and accelerated cell proliferation and migration in HCC cases, respectively [21,22,23]. Zhang et al., reported that in patients with HCV-related HCC, the expression of lncRNA-HEIH was increased in both serum and exosomes with a higher level in exosomes than serum [24].

Other lncRNAs, such as LINC02394 and LINC00635, were reported to be associated with portal vein tumor thrombus, lymph node metastasis, TNM stage, and overall survival (OS). The combination of lncRNAs and serum AFP levels is useful for diagnosing HCC and predicting the prognosis of HCC [25]. All these findings support the role of lncRNAs in hepatocarcinogenesis.

A variety of cancers have been associated with long Intergenic Non-Protein Coding RNA 1564 (LINC01564) that is closely related to glutamate-cysteine ligase catalytic subunit [26]. At least four different splicing isoforms of the gene linc01564 have been expressed, the longest isoform, linc01564-1, was recently given the name RAMS11. There are 148 differentially expressed RNAs Associated with Metastasis (RAMS). In vitro and in vivo studies have shown that RAMS11 promotes aggressive phenotypes and is linked to poor disease-free survival [27].

The protein-coding gene chromobox 4 (Cbx4), which has six exons and spans about 6.26 kb, is located on chromosome 17q25. According to recent evidence, the dysregulation of this gene may alter the carcinogenic process of several tumors, including HCC, colorectal cancer, breast cancer, and others, and may be an important prognostic biomarker [28].

DNA topoisomerase is an enzyme present in the nuclei of living organisms and located on chromosome 17. It alters the three-dimensional structure of DNA and catalyzes the modification of DNA topologies by binding and breaking of DNA strands. DNA topoisomerase type I (TOP1) breaks the single strand, while DNA topoisomerase II (TOP2) slices off the double chain [29]. The transcription and synthesis of DNA, as well as chromosome segregation during mitosis, depend on TOP2A. The cell cycle and cell proliferation are linked to its expression [30].

The aim of this study is to detect if there is an association between expression of some long non coding RNAS (LNCRNAs) as (RAMS11, LINC01564), CBX4, and TOP2A and clinical; pathological characteristic of hepatocellular carcinoma in Egyptian patients.

## 2. Materials and Methods

This research was done by cooperation between, Department of biochemistry, Menoufia University’s Faculty of Science, Molecular biology and Medical Biochemistry, Medical Microbiology & Immunology, Clinical Oncology and Internal Medicine Departments, Menoufia University’s Faculty of Medicine between December 2021 and May 2022. It was conducted on 150 subjects classified into 3 groups: Group I consisted of 50 patients who had hepatocellular carcinoma on top of chronic hepatitis C, Group II consisted of 50 patients who had chronic hepatitis C, and Group III consisted of 50 people who appeared to be in good health.

The approval from the Ethics Committees, Faculty of Medicine, Menoufia University was taken. All participants signed informed written consents. All participants underwent complete history taking and thorough clinical examination. All patients were subjected to abdominal ultrasonography using probe 3.5 MHZ of TDI Philips machine. Patients with HCC were diagnosed by triphasic computerized tomography (CT). Patients were subjected to baseline chest, abdomen and pelvis CT, and bone scans for the assessment of distant metastases.

The Barcelona Clinic’s staging system for liver cancer and the Child-Pugh classification were used to evaluate the clinical staging of HCC. Clinicopathological features were received at the moment of blood collection for HCC cases, including tumor number, size, location, evidence of metastasis, and portal vein thrombosis. Patients with chronic HBV infection or any other chronic hepatitis-causing condition besides HCV were disqualified from the research after making hepatitis markers. The Child-Pugh score, which considers five standards clinical (hepatic encephalopathy and ascites) and laboratory (albumin, prothrombin time, and bilirubin levels) indicators was used to determine the severity of the disease [31].

### 2.1. Collection of Samples and Laboratory Tests

By using a sterile venipuncture, ten milliliters (mL) of peripheral blood were obtained. To prepare two milliliters for real-time PCR and DNA purification, they were moved to tubes containing EDTA. Using a Sysmex XN-1000 (Japan (19723), B.M. Egypt firm), two ml of blood in EDTA-containing tubes was used for a complete blood count (CBC). Then, the plasma obtained from centrifugation of this tube was then used for lncRNA extraction and real-time qPCR. The resultant sera from four ml of blood transferred to an empty test tube were stored frozen at 20 °C until use after being centrifuged at 4000 rpm for 10 min. According to the manufacturer’s instructions, the AFP levels in the serum were measured using enzyme-linked immunosorbent assay (ELISA) kits from Thermo Fisher Scientific, USA, and Leinco Technologies, USA, respectively. A kinetic UV-optimized approach, IFCC with an ANDOX Kit, UK, was used to measure the enzymes aspartate aminotransferase (AST) and alanine aminotransferase (ALT). A kit provided by Diamond Diagnostics, Germany, was used to assess the amounts of albumin, total bilirubin, and creatinine using a standard colorimetric approach. Viral markers (HCVAb–HbsAg) were determined by electrochemiluminescence using the Cobase immunoassay analyzer and the immunoassay “ECLA” (Roche Diagnostic, Germany). A citrate tube was used to collect 2 mL of fresh blood for the purpose of prothrombin time (PT) determination (BIOMED- LlQUIPLASTIN diagnostic kit, Germany).

### 2.2. Quantitative Real-Time Polymerase Chain Reaction (qRT-PCR) for LncRNAs (RAMS11, Linc01564), CBX4, and TOP2A Genes as a Target and GAPDH Gene as an Endogenous Reference Gene

Using the miRNeasy isolation kit, total RNA was extracted from whole blood for gene expression and 100 μL of fresh plasma samples for lncRNA extraction (QIAGEN, Hilden, Germany). A tool called a Nanodrop was used to measure RNA concentration—and quality (Thermo Scientific, Philadelphia, PA, USA). To create complementary DNA, reverse transcription was done using the miScript II RT Kit from QIAGEN (cDNA). Each reaction was conducted in a 20 μL total volume on ice: 4 μL miScript High-Specific-Rate Buffer, 2 μL miScript Nucleic Mix, 2 μL Reverse transcriptase. The mixture was pipetted into each well, along with 2 μL of nuclease-free water. Each PCR tube received a pipette of the mixture. The extract was then pipetted into each tube in a volume of 10 μL. Singapore 2720, Applied Biosystems, thermal cycler was used for incubation for a single cycle at 37 °C for 60 min and 95 °C for 5 min. The generated cDNA was kept at −20 °C until the real-time PCR phase. The SYBR Green PCR Kit was used to perform real-time PCR (QIAGEN). A total volume of 25 μL was used before amplification, consisting of 12.5 μL of SYBR green Master Mix (QIAGEN) with low ROX, 3.5 μL of nuclease-free water, 4 μL of diluted cDNA, and 2.5 μL of forward and reverse primer for each gene with a specified sequence as follows: CBX4 primer sequences (F primer: CTGGTGAAATGGAGAGGC—R primer: GAACGACGGGC AAAGGTAGG), TOP2A primers (Forward primer: CTAGTTAATGCTGCGGACAACA, Reverse primer: CATTTCGACCACCTGTCACTT), RAMS11 primers (F primer: TCCACTT CCAGCAAGGGATG, R primer: TTGGGGCGAGGACCATCTAT)—Linc01564 primers (Forward primer: CAGCTGGCTGAAGAGCTCAA, Reverse primer: GTTACTGCAGTCCCTTGGGG), GAPDH primers (reference gene) Forward CTCTGCTCCTCCTGTTCGAC Reverse TTAAAAGCAGCCCTGGTGAC. Data was analyzed using the 2.0.1 version of the Applied Biosystems 7500 software. The comparative ΔΔ Ct method was used to perform relative quantification (RQ) of gene expression, where the amount of the target genes was normalized to an endogenous reference gene (GAPDH) and relative to a control.

**Figure 1:**
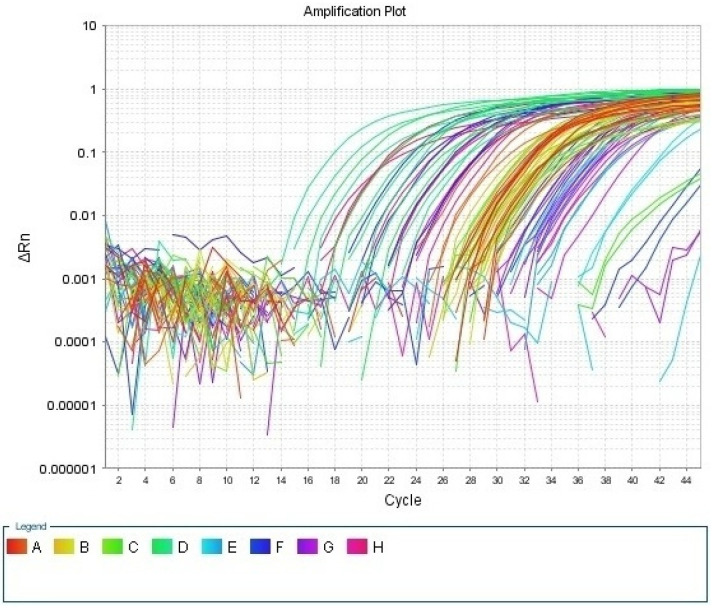
Amplification plot for the first run.

**Figure 2:**
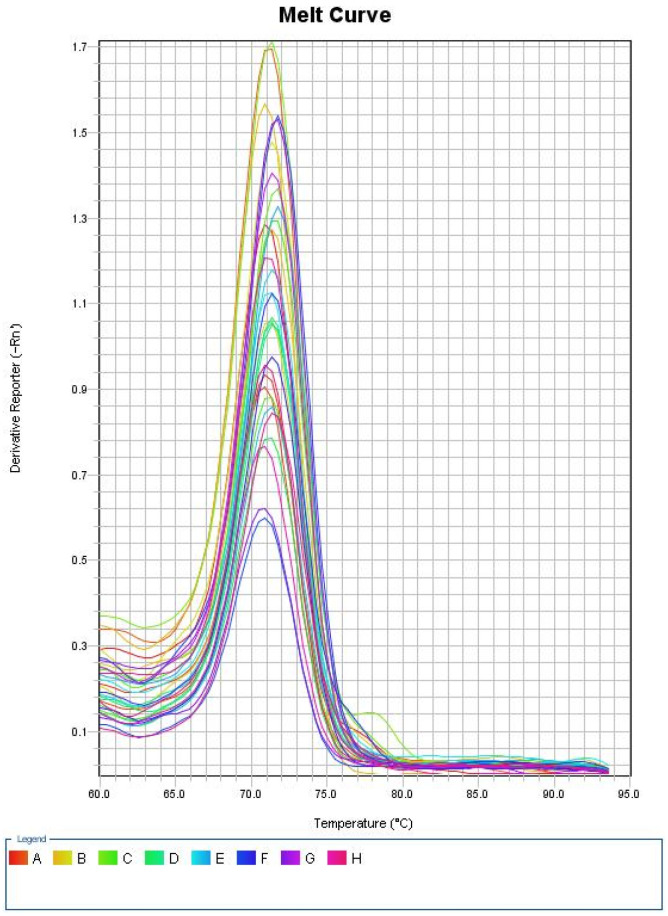
Melting curve for the first run.

Melting curve analysis was used to finish each run and verify the specificity of the amplification and lack of primer dimers. Figure 1 showed amplification plot for the first run, and Figure 2 showed melting curve for the first run.

### 2.3. Statistical Analysis of the Data

With the aid of the IBM SPSS software package version 20.0, data were fed into the computer and evaluated (Armonk, NY, USA: IBM Corp). The Kolmogorov-Smirnov test was used to ensure that the distribution of the variables was normal; Chi-square test was employed to compare two groups for categorical variables. Alternatively, Fisher Exact correction test was performed when more than 20% of the cells have expected count less than 5. ANOVA was used to compare the three study groups and followed by Post Hoc test (Tukey) for pairwise comparison while for abnormally distributed quantitative data, the Kruskal Wallis test was used to compare different groups and followed by Post Hoc test (Dunn’s for multiple comparisons test) for pairwise comparison. The Spearman coefficient was used to correlate between quantitative variables. Univariate and multivariate regression analyses were performed to calculate the effects of risk factors as independent. A receiver operating characteristic curve (ROC) was utilized. Significance of the obtained results was judged at the 5% level.

## 3. Results

### 3.1. Clinical and Biochemical Characteristics of the Studied Groups

Sex and age were insignificantly different among the three groups. ALT, AST, total bilirubin and direct bilirubin were significantly increased in HCV and HCC compared to control group (*p* value < 0.001). In contrast, platelet count, Hb and serum albumin was significantly decreased in HCV and HCC compared to control group (*p* value < 0.001). α FP was significantly increased in HCC group compared to HCV and control groups (*p* value < 0.001), and in HCV group compared to control group (Table 1). There was insignificant difference between HCC and HCV groups in loss of weight, jaundice, hepatic encephalopathy, splenomegaly and comorbidities. Ascites and Child Pugh Class was significantly decreased in HCC compared to HCV group (Table 2). Regarding TNM staging, 7 (14%) patients were presented with stage I tumor, 3 (6%) were presented with stage II tumor, 19 (38%) patients were presented with stage IIIa tumor, 9 (18%) patients were presented with stage IIIb tumor, 1 (2%) patient had stage IIIc tumor, 11 (22%) patients were presented with stage IVa tumor. Regarding outcome, 15 (30%) of the studied patients died. The overall survival time of the studied patients ranged from 6–24 months with a mean value of 20.3 ± 5.9 months.

### 3.2. The Relative Expression Level of lncRNA in Studied Groups

This study was considered the first to investigate the relative expression level of these types of lncRNA in Egyptian patients infected with HCV to find an early and non-invasive biomarker for HCC. There were significant high values in the case group. Long noncoding RNAs (Linc01564, RAMS11), CBX4, TOP2A were significantly higher in HCC group compared to HCV and control groups (*p* value < 0.001) and significantly higher in HCV group when compared to control group (Table 3).

The diagnostic performance of the relative expression level of (Linc01564, RAMS11), CBX4, TOP2A in discriminating HCC group from HCV group.

**Figure 3:**
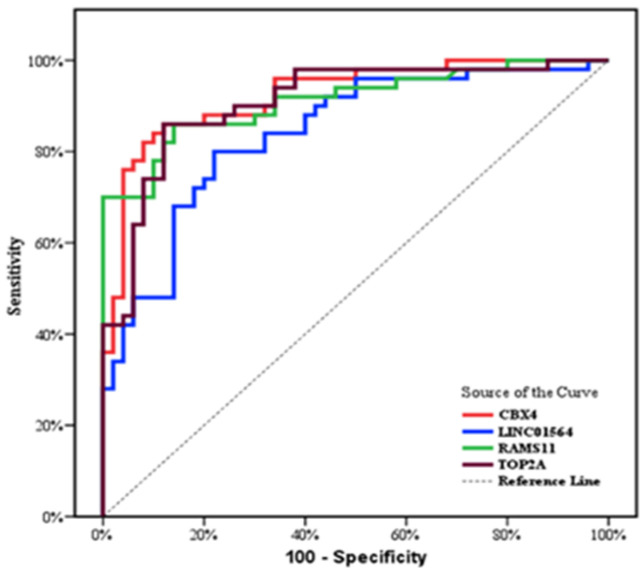
ROC curve for different parameters to discriminate HCC group form HCV group.

LINC01564, RAMS11, CBX4 and TOP2A can significantly discriminate HCC patients from HCV group (*p* < 0.001) with high sensitivity and specificity for each of them (Table 4). The power of the relative expression levels of lncRNA to diagnose HCC from CHC cases was evaluated using ROC analysis (Figure 3).

### 3.3. Regarding Correlations between the Relative Expression Level of lncRNA with Clinical and Biochemical Parameters among Case Group Showed That

The relative expression level of lncRNA (CBX4, LINC01564, RAMS11 and TOP2A) showed a positive correlation in HCC group with ALT, AST and overall survival time, on the other hand were insignificantly correlated with age, Hb, platelets, WBCS, serum albumin, total bilirubin, direct bilirubin, PT and α FP (Table 5). 

### 3.4. Linear Regression Analysis in the HCC Group Revealed That 

In univariate regression analysis, total bilirubin, direct bilirubin, α FP, BCLC(C), CBX4, RAMS11 were significant predictors for mortality. In multivariate regression analysis, LN metastasis, LINC01564 and TOP2A were independent predictor for mortality (*p* value = 0.044, HR = 13.077 (1.065–160.525)) and (*p* value = 0.038, HR = 1.544 (1.024–2.329)), respectively (Table 6).

## 4. Discussion

HCC is caused mainly by cirrhosis, bilharziasis, hepatitis B or C virus infections, alcoholism, smoking, chemical exposure, and poisons like aflatoxin [32]. Chronic hepatitis C virus (HCV) infection, the third most common cause of HCC, is responsible for around one-third of all incidence rates and one-fifth of HCC-related deaths [33]. Compared to healthy persons, those with HCV have an approximately 17-fold increased risk of developing HCC [34]. Other studies investigated that the reason why HCV is a major risk factor associated with HCC, is the progression of fibrosis [35,36]. Bruno et al., have demonstrated that HCV can cause alterations in the glucose and lipid metabolism and stimulate insulin-like growth factors, which in turn causes the stellate cells in the liver to become active with subsequent development of fibrosis [37].

Despite the fact that cirrhosis and changes in cell regeneration mechanisms are the main pathogenic factors of HCV-related HCC, alterations in gene expression and various signal transduction pathways have also been described as being involved in the proliferation and malignant transformation of hepatocytes in chronic HCV infection [38]. Alpha-fetoprotein (AFP) is considered the most commonly used tumor marker for the identification and monitoring of HCC, but it is of low specificity and elevated also in non-cancer liver diseases such cirrhosis and chronic hepatitis [39]. Biomarkers need to have high sensitivity and specificity for HCC, reflect changes in tumor prognosis and progression, and be simple and non-invasive to detect in bodily fluids in order to be therapeutically helpful [40].

Accumulating evidence supports the fact that deregulated lncRNA expression may contribute to cellular proliferation and invasion through numerous mRNA, growth signal proteins and invasion markers. These circulating lncRNAs have been identified as promising biomarkers for the diagnosis and prognosis of HCC [6].

The most important findings of the present study were that the relative expression levels of circulatory lncRNA (LINC01564, RAMS11), CBX4, and TOP2A were upregulated in CHC groups. Furthermore, the highest relative expression level was in HCC patients compared to CHC. These findings are in line with those of Zheng et al., who found that lncRNA RAMS11 expression was upregulated in tissue samples of prostate cancer. They also investigated whether lncRNA RAMS11 bound to CBX4 to activate the expression of Top2, and they found that this binding to Top2 increased prostate cancer cell growth and metastasis [41].

Wang et al., reported that there was an increase in TOP2A in HCC tissues, which was associated with the T and M stages as well as the proliferation, migration, and invasion capacities of HCC cells both in vitro and in vivo. Additionally, they showed that blocking TOP2A prevented tumor development and metastasis in vivo as well as in vitro migration and invasion of HCC cells [42].

Our findings showed that the level of LINC01564 was markedly greater in patients with LN metastasis compared to those without LN metastasis (*p* value 0.032). Also, LINC01564 and CBX4 were significant higher in patients with IV stage tumor than those with I, II and III. This is consistent with a study conducted in malignant disease who found that Cbx4 expression was elevated in HCC tissues, and Cbx4 overexpression was associated to tumor size, pathologic differentiation, and TNM (tumor, node, metastasis) phases as well as the blood level of alpha-fetoprotein (AFP) [3]. 

Regarding Top2A and RAMS11, they had the same results as CBX4, LINC01564. Panvichian et al., demonstrated that there was no significant association between age, tumor size, AFP, or TP53 and TOP2A overexpression [43]. According to Watanuki et al., overexpression of TOP2A in HCC is thought to be associated with a potentially aggressive tumor phenotype and cancer-related mortality and that the predictive value of TOP2A overexpression in HCC is statistically significant [44]. Also, Cai et al., investigated that increased expression of TOP2A in HCC was correlated with an advanced clinical stage, a low grade of tumor differentiation and a high T stage [45]. 

Multivariate regression analysis, in the current study, stated that LN metastasis, LINC01564 and TOP2A were independent predictor for mortality, and in agreement with our results, Meng and his coworkers confirmed that in a multivariate analysis, the TOP2A overexpression was an independent indicator of unfavorable overall survival in HCC after adjusting other prognostic indicators [46]. 

EMT (Epithelial to Mesenchymal Transition) has been increasingly recognized to occur during the progression of various carcinomas such as hepatocellular carcinoma (HCC). It was discovered that TOP2A may promote EMT, which is mediated by the p-ERK1/p-SMAD2 (S425/250/255)/Snail signaling pathway, and increase HCC cell migration and invasion [47]. Furthermore, TOP2A overexpression in HCC has been linked to earlier age of onset, shorter survival periods, and resistance to doxorubicin-based treatment, according to Wong et al., Therefore, there is an urgent need for new, more effective TOP2A-targeted chemotherapeutic drugs [48].

ROC curve analysis reveals the power of the relative expression levels of LINC01564, RAMS11, CBX4 and TOP2A in discriminating HCC patients from HCV group, suggesting the possible role of these molecular non-invasive markers in early diagnosis of HCC in chronic hepatitis C cases. These findings are consistent with earlier research, which have showed that the expression levels of TOP2A are elevated in a variety of cancers, including colorectal [49], liver [50], esophageal [51], and gastric [52], and that TOP2A may be used as a biomarker to screen out high-risk cases and forecast the prognosis of patients with malignant tumors in order to enable tailored treatment [53].

## 5. Conclusions

The results of the current study have showed that the relative expression level of lncRNAs (RAMS11, LINC01564), CBX4, and TOP2A were upregulated in CHC patients and more in CHC patients with HCC, suggesting a promising non-invasive diagnostic role of these markers for HCC especially TOP2A.

## Figures and Tables

**Table 1:** Demographic and laboratory data in the three studied groups.

	HCC (n = 50)	HCV (n = 50)	Control (n = 50)	Test of Sig.	*p*
**Age (years)**					
Mean ± SD.	59.5 ± 5.7	58.1 ± 3.3	59.7 ± 4	F = 1.874	0.157
**Gender**					
Male	37 (74%)	36 (72%)	31 (62%)	χ^2^ = 1.944	0.378
Female	13 (26%)	14 (28%)	19 (38%)		
**ALT (IU/L)**					
Median (Min.–Max.)	38 (11–76)	50 (6–168)	22 (9–47)	H = 45.50 *	<0.001 *
**Sig. bet. Groups**	*p*_1_ = 0.384, *p*_2_ < 0.001 *, *p*_3_ < 0.001 *				
**AST (IU/L)**					
Median (Min.–Max.)	40.5 (16–78)	52 (8–138)	23 (9–50)	H = 60.234 *	<0.001 *
**Sig. bet. Groups**	*p*_1_ = 0.428, *p*_2_ < 0.001 *, *p*_3_ < 0.001 *				
**Hb (g/dl)**					
Mean ± SD.	10.69 ± 1.41	12.54 ± 1.33	12.96 ± 0.84	F = 48.796 *	<0.001 *
**Sig. bet. Groups**	*p*_1_ < 0.001 *, *p*_2_ < 0.001 *, *p*_3_ = 0.198				
**Platelets (×10³/UL)**					
Mean ± SD.	126.5 ± 53.3	173.6 ± 76.7	266.9 ± 66.3	F = 58.421 *	<0.001 *
**Sig. bet. Groups**	*p*_1_ = 0.001 *, *p*_2_ < 0.001 *, *p*_3_ < 0.001 *				
**WBCs (×10³/UL)**					
Mean ± SD.	5.8 ± 1.6	6.6 ± 1.9	5.9 ± 1.9	F = 2.670	0.073
**Serum albumin (g/dl)**					
Median (Min.–Max.)	3.2 (2.3–3.9)	3.4 (2.3–4.4)	4.5 (3.7–5)	H = 93.732 *	<0.001 *
**Sig. bet. Groups**	*p*_1_ = 0.007 *, *p*_2_ < 0.001 *, *p*_3_ < 0.001 *				
**Total Bilirubin (mg/dl)**					
Median (Min.–Max.)	1.8 (0.4–15.7)	0.8 (0.2–5)	0.6 (0.2–1)	H = 63.090 *	<0.001 *
**Sig. bet. Groups**	*p*_1_ < 0.001 *, *p*_2_ < 0.001 *, *p*_3_ < 0.001 *				
**Direct bilirubin (mg/dl)**					
Median (Min.–Max.)	0.7 (0.1–11.2)	0.2 (0.1–2)	0.2 (0.1–0.3)	H = 70.297 *	<0.001 *
**Sig. bet. Groups**	*p*_1_ < 0.001 *, *p*_2_ < 0.001 *, *p*_3_ = 0.019 *				
**PT (%)**					
Median (Min.–Max.)	84.8 (60–95)	81 (56–97.3)	98.5 (94–101)	H = 98.201 *	<0.001 *
**Sig. bet. Groups**	*p*_1_ = 0.496, *p*_2_ < 0.001 *, *p*_3_ < 0.001 *				
**α FP (ng/mL)**					
Median (Min.–Max.)	123.5 (7–2600)	23.4 (2.1–614)	3 (1–6)	H = 100.311 *	<0.001 *
**Sig. bet. Groups**	*p*_1_ < 0.001 *, *p*_2_ < 0.001 *, *p*_3_ < 0.001 *				

SD: Standard deviation ALT: Alanine transaminase, AST: Aspartate transaminase, Hb: hemoglobin, WBCS: white blood cells, PT: prothrombin time, α FP: Alpha 1- fetoprotein. F: F for ANOVA test, pairwise comparison bet. each 2 groups were done using Post Hoc Test (Tukey). H: H for Kruskal Wallis test, pairwise comparison bet. each 2 groups were done using Post Hoc Test (Dunn’s for multiple comparisons test). *p*: *p* value for comparing between the studied groups. *p*_1_: *p* value for comparing between HCC and HCV. *p*_2_: *p* value for comparing between HCC and Control. *p*_3_: *p* value for comparing between HCV and Control. *: Statistically significant at *p* ≤ 0.05.

**Table 2:** Clinical data in the two patients’ groups (HCC & HCV).

	HCC (n = 50)	HCV (n = 50)	χ^2^	*p*
**Loss of Weight**	34 (68%)	34 (68%)	0.0	1.000
**Jaundice**	13 (26%)	11 (22%)	0.219	0.640
**Hepatic Encephalopathy**	10 (0%)	4 (8%)	2.990	0.084
**Splenomegaly**	34 (68%)	32 (64%)	0.178	0.673
**Ascites**				
No	12 (24%)	19 (38%)	10.082 *	0.018 *
Minimal	21 (42%)	23 (46%)		
Moderate	6 (12%)	7 (14%)		
Tense	11 (22%)	1 (2%)		
**Comorbidity**				
DM	14 (28%)	21 (42%)	2.154	0.142
HTN	16 (32%)	16 (32%)	0.0	1.000
Heart Disease	1 (2%)	1 (2%)	0.0	*^FE^ p* = 1.000
**Child Pugh Class**				
A	12 (24%)	19 (38%)	6.422 *	0.040 *
B	25 (50%)	27 (54%)		
C	13 (26%)	4 (8%)		

χ^2^: Chi square test; MC: Monte Carlo; *^FE^*: Fisher Exact. *p*: *p* value for comparing between the studied groups. *: Statistically significant at *p* ≤ 0.05.

**Table 3:** Long noncoding RNAs (LNCRNAs) in the three studied groups.

	HCC (n = 50)	HCV (n = 50)	Control (n = 50)	H	*p*
**CBX4**					
Median (Min.–Max.)	1.882 (0.180–8.900)	0.300 (0.007–2.915)	0.062 (0–1.035)	96.277 *	<0.001 *
**Sig. bet. Groups**	*p*_1_ < 0.001 *, *p*_2_ < 0.001 *, *p*_3_ < 0.001 *				
**LINC01564**					
Median (Min.–Max.)	2.081 (0.117–12.040)	0.431 (0.002–4.158)	0.056 (0–1.024)	95.376 *	<0.001 *
**Sig. bet. Groups**	*p*_1_ < 0.001 *, *p*_2_ < 0.001 *, *p*_3_ < 0.001 *				
**RAMS11**					
Median (Min.–Max.)	1.872 (0.172–9.520)	0.381 (0.000–1.306)	0.049 (0.001–1.003)	99.748 *	<0.001 *
**Sig. bet. Groups**	*p*_1_ < 0.001 *, *p*_2_ < 0.001 *, *p*_3_ < 0.001 *				
**TOP2A**					
Median (Min.–Max.)	1.932 (0.050–10.800)	0.269 (0.000–2.400)	0.046 (0.001–1.009)	91.454 *	<0.001 *
**Sig. bet. Groups**	*p*_1_ < 0.001 *, *p*_2_ < 0.001 *, *p*_3_ < 0.001 *				

*p*_1_: *p* value for comparing between HCC and HCV. *p*_2_: *p* value for comparing between HCC and Control. *p*_3_: *p* value for comparing between HCV and Control. *: Statistically significant at *p* ≤ 0.05.

**Table 4:** Diagnostic performance of different parameters to discriminate HCC group form HCV group.

	AUC	*p*	95% C.I	Cut off	Sensitivity	Specificity	PPV	NPV
**LINC01564**	0.84	<0.001	0.763–0.917	>1.3046	80	78	78.4	79.6
**RAMS11**	0.911	<0.001	0.854–0.969	>1.006	86	86	86	86
**CBX4**	0.923	<0.001	0.871–0.975	>1.012	86	88	87.8	86.3
**TOP2A**	0.908	<0.001	0.850–0.967	>1.0521	82	88	87.2	83

**Table 5:** Correlation between different gene expression with laboratory data and overall survival in HCC group (n = 50).

		CBX4	LINC01564	RAMS11	TOP2A
Age (years)	r_s_	0.081	0.093	0.109	−0.070
	*p*	0.576	0.522	0.451	0.630
ALT	r_s_	0.366	0.341	0.381	0.354
	*p*	0.009	0.015	0.006	0.012
AST	r_s_	0.362	0.305	0.373	0.334
	*p*	0.010	0.031	0.008	0.018
Hb	r_s_	0.156	0.025	0.052	0.028
	*p*	0.280	0.861	0.719	0.850
Platelets	r_s_	−0.141	0.056	0.030	−0.080
	*p*	0.329	0.697	0.835	0.580
WBC_S_	r_s_	0.013	0.061	0.043	−0.138
	*p*	0.928	0.673	0.768	0.340
Serum albumin	r_s_	−0.186	−0.139	0.055	−0.096
	*p*	0.195	0.337	0.702	0.507
Total Bilirubin	r_s_	0.011	0.043	−0.008	0.120
	*p*	0.938	0.768	0.957	0.405
Direct bilirubin	r_s_	0.027	0.042	−0.006	0.148
	*p*	0.854	0.771	0.966	0.305
PT	r_s_	0.106	−0.106	−0.107	−0.104
	*p*	0.463	0.463	0.458	0.471
α FP	r_s_	0.068	−0.007	−0.037	0.077
	*p*	0.638	0.963	0.800	0.597
Overall survival time (months)	r_s_	−0.470	−0.572	−0.622	−0.573
	*p*	0.001	<0.001	<0.001	<0.001

**Table 6:** Univariate and multivariate COX regression analysis for the parameters affecting mortality.

	Univariate		^#^ Multivariate	
	*p*	HR (LL–UL 95% C.I)	*p*	HR (LL–UL 95% C.I)
Age (years)	0.511	1.032 (0.939–1.134)		
Gender (female)	0.446	0.611 (0.172–2.168)		
ALT	0.108	1.026 (0.994–1.059)		
AST	0.294	1.016 (0.986–1.046)		
Hb	0.370	0.837 (0.567–1.235)		
Platelets	0.378	1.004 (0.995–1.013)		
WBCS	0.476	0.878 (0.614–1.255)		
Serum albumin	0.695	1.240 (0.423–3.635)		
Total Bilirubin	0.017 *	1.143 (1.024–1.275)	0.733	1.296 (0.293–5.727)
Direct bilirubin	0.024 *	1.185 (1.023–1.373)	0.986	0.982 (0.127–7.621)
PT	0.592	1.017 (0.957–1.080)		
α FP	0.032 *	1.001 (1.0–1.001)	0.444	1.000 (0.999–1.001)
Vascular invasion	0.262	1.852 (0.631–5.437)		
LN metastasis	0.023 *	3.259 (1.176–9.032)	0.044 *	13.077 (1.065–160.525)
Tumor Number	0.751	1.204 (0.383–3.781)		
Tumor Size	0.529	1.386 (0.502–3.822)		
TNM Staging (III + IV)	0.189	30.688 (0.186–5072.267)		
BCLC (C)	0.003 *	5.556 (1.759–17.550)	0.786	0.711 (0.061–8.323)
CBX4	0.002 *	1.299 (1.101–1.533)	0.524	0.864 (0.552–1.354)
LINC01564	<0.001 *	1.345 (1.165–1.553)	0.035 *	1.297 (1.019–1.651)
RAMS11	<0.001 *	1.385 (1.184–1.620)	0.730	0.916 (0.555–1.510)
TOP2A	<0.001 *	1.580 (1.301–1.919)	0.004 *	2.543 (1.345–4.809)

HR: Hazard ratio; C.I: Confidence interval; LL: Lower limit; UL: Upper Limit. ^#^: All variables with *p* < 0.05 was included in the multivariate. *: Statistically significant at *p* ≤ 0.05.

## Data Availability

Not applicable.
